# The Fumigation Toxicity of Three Benzoate Compounds against Phosphine-Susceptible and Phosphine-Resistant Strains of *Rhyzopertha dominica* and *Sitophilus oryzae*

**DOI:** 10.3390/insects15070477

**Published:** 2024-06-27

**Authors:** Md Munir Mostafiz, Hwal-Su Hwang, Jun-Ran Kim, Bong-Su Kim, Kyeong-Yeoll Lee

**Affiliations:** 1 Department of Plant Medicine, College of Agriculture and Life Sciences, Kyungpook National University, Daegu 41566, Republic of Korea; munir93@knu.ac.kr (M.M.M.); bgtwo2@knu.ac.kr (H.-S.H.); 2 Institute of Agricultural Science and Technology, Kyungpook National University, Daegu 41566, Republic of Korea; 3 Institute of Plant Medicine, Kyungpook National University, Daegu 41566, Republic of Korea; 4 Plant Quarantine Technology Center, Animal and Plant Quarantine Agency (APQA), Gimcheon 39660, Republic of Korea; junrankim@korea.kr (J.-R.K.); bskim79@korea.kr (B.-S.K.)

**Keywords:** fumigation, natural pesticides, stored-product insects, phosphine-resistance, quarantine

## Abstract

**Simple Summary:**

Many species of insects that infest stored products have developed a resistance to phosphine as a result of its widespread use to control stored-product pests. There is a significant opportunity for the development of natural fumigants as eco-friendly methods for controlling pests in stored products. A plant-derived molecule called methyl benzoate (MBe) is an example of a volatile organic compound that has potent insecticidal and fumigation toxicity. In this research, we examined its potential efficacy as a fumigation agent against phosphine-resistant strains of the lesser grain borer and rice weevil.

**Abstract:**

Phosphine (PH_3_) has been widely used as a fumigant in food storage, but increasing PH_3_ resistance in major pests makes finding alternative fumigants urgent. Methyl benzoate (MBe), a volatile organic compound regarded to be a food-safe natural product, has recently demonstrated significant toxicity against a variety of insect pests. This study is the first evaluation of the fumigation toxicity of three benzoate compounds, MBe, vinyl benzoate, and ethyl benzoate, against PH_3_-susceptible and PH_3_-resistant strains of *Rhyzopertha dominica* and *Sitophilus oryzae*. All strains were exposed to the compounds at concentrations up to 20 µL/1.5 L air for 24 h. Compared to vinyl benzoate and ethyl benzoate, MBe induced higher mortality rates in all strains at all concentrations. When food was made available, the lethal median concentration for MBe was 10–17-fold higher than when tested without food. Moreover, no significant differences were observed between the responses of the PH_3_-susceptible and PH_3_-resistant strains to the compounds. Notably, *S. oryzae* was more susceptible to MBe. In laboratory settings, MBe successfully controlled PH_3_-resistant strains of *R. dominica* and *S. oryzae*, making it a viable option for PH_3_-resistance management. Thus, MBe might be suitable for food security programs as an environmentally benign alternative fumigant.

## 1. Introduction

Many stored-product and post-harvest insects have the potential to damage a wide range of stored and produced agricultural products [1]. In addition to the direct loss of stored grain due to pest infestation [2], commodities and packaged foods [3] may become contaminated with allergens and arthropod corpses as a consequence of pest infestation [4]. Currently, the risks associated with storage pests are increasing as a result of climate change [5,6]. While this makes it more likely that commodities will become infested while being transported internationally [7], it has also led to pests becoming more resistant to a wide variety of pesticides [8]. Thus, increasing postharvest commodity degradation as a result of climate change occurs while moving through the agricultural supply chain [9]. Consequently, pest control operators, food industry managers, farmers, grain storekeepers, and commodities merchants face a significant challenge when it comes to the effective control and management of storage pests.

In the bulk storage of commodities, fumigation is the most prevalent method of post-harvest pest control [10]. Throughout history, methyl bromide (MB) and phosphine (PH_3_) have been the most frequently used fumigants [11]. However, MB has been phased out of use in the majority of applications because it contributes to atmospheric ozone-layer depletion [12,13]. Although PH_3_ is still extensively used as the primary post-harvest fumigant, there have been growing concerns over its usage [14]. For instance, at least six different taxa of stored product pests have shown significant increases in PH_3_-resistant populations globally [15]. This phenomenon may occur when the appropriate dosage of PH_3_ is surpassed, resulting in an unexpected increase in insect survival rather than a decline [16]. The primary assessment procedure for measuring the prevalence of PH_3_ resistance is the Food and Agriculture Organization’s (FAO’s) approach, which relies on exposures typically lasting 20 h and concentrations of about 30 parts per million (ppm) [17,18]. After a population is identified as resistant, a selected sample of individuals from that group is exposed to various concentrations of PH_3_, ranging from low to high, and the relationship between the dosage and the death rate is then analyzed using probit regression [17]. Then, the PH_3_ resistance is classified as either weak or strong.

While various alternative fumigants, such as sulfuryl fluoride [19], ethyl formate [20], hydrogen cyanide [21], and nitric oxide [22], have been examined by researchers, these fumigants have several drawbacks [21,23]. Because of this, there is a growing movement to use pest control strategies that are less toxic and less harmful to the environment, which has led to an interest in naturally occurring toxins, like plant essential oils and their main components, instead of synthetic pesticides in pest control programs.

Naturally occurring toxins tend to break down quickly in the environment and are less toxic to non-target organisms, such as the pests’ natural enemies, humans, and other vertebrates [24,25]. According to Feng et al. [26], benzoate chemicals, plant derivatives that have the potential to suppress insect pests and mites, include methyl benzoate (MBe), ethyl benzoate (EB), and vinyl benzoate (VB). As a result of its low toxicity to humans and the fact that it has been authorized for use as an ingredient in foods by both the Food and Drug Administration of the United States and the European Union. MBe is a viable alternative pesticide that may be used to manage agricultural, urban, and medical pests [27,28,29,30]. Additionally, MBe is classified as harmless to both predators and pollinators by the International Organization for Biological Control [31,32,33,34,35]. Recently, MBe has been identified as an effective alternative fumigating agent in the management of bed bugs and some insects that infest stored products [28,30,36,37,38]. However, there is a lack of information about its use in the management of PH_3_-resistant stored-product insects.

This research focused on two stored-product insects that are resistant to PH_3_, namely the lesser grain borer (*Rhyzopertha dominica* Fabricius) and the rice weevil (*Sitophilus oryzae* Linnaeus). The lesser grain borer is a significant primary pest that infests entire grain kernels in large storage conditions [39], while the rice weevil is a widespread pest that affects stored grain and has developed considerable resistance to PH_3_ [40]. The PH_3_ resistance of both has significantly increased worldwide, demanding the use of resistance management strategies. Hence, the primary aim of this investigation was to examine the fumigation toxicities of three benzoate derivatives, MBe, EB, and VB, against PH_3_-resistant and PH_3_-susceptible *R. dominica* and *S. oryzae* strains and compare the results. Furthermore, we also examined the fumigation toxicity of MBe on these two species under feeding conditions.

## 2. Materials and Methods

### 2.1. Insects

The PH_3_-susceptible strain and the PH_3_-resistant strain of *R. dominica* were obtained from Murdoch University in Western Australia [41]. The PH_3_-susceptible strain of *S. oryzae* was obtained from Murdoch University, whereas the PH_3_-resistant strain was raised at Chungbuk National University (Chungcheongbuk-do, Republic of Korea) [42]. All the tested insects were taken to the Plant Quarantine Technology Center (PQTC) in Gimcheon, Korea, and continued to be reared there. For the experiments, insects from the PQTC were brought to Kyungpook National University in Korea to be raised. The rearing conditions varied by species: *R. dominica* was raised on a combination of oatmeal and wheat flour in a ratio of 80:20, while *S. oryzae* was raised on rice grains in 500 mL glass jars. Both insects were raised in controlled laboratory conditions at 26 ± 1 °C, at a relative humidity of 60–70%, and using a light–dark photoperiod of 14:10 h.

### 2.2. Chemicals

We acquired PH_3_ in the form of ECO2Fume, which consists of 2% PH_3_ and 98% CO_2_, from Cytec, located in Sydney, NSW, Australia. Both MBe and EB (99% purity) were acquired from Sigma-Aldrich in St. Louis, MO, USA, while VB (99% purity) was obtained from Tokyo Chemical Industry Co., Ltd. in Toshima, Kita-Ku, Tokyo, Japan.

### 2.3. PH_3_-Resistance Assessment

Treatment with PH_3_ was performed at the PQTC. The PH_3_ fumigation process was carried out in a 12 L glass desiccator manufactured by DWK Life Sciences in Mainz, Germany. The fumigation chamber was set to a temperature of 20 ± 1 °C and a relative humidity of 60 ± 10%. A total of thirty adult insects were placed in a 100 × 40 mm insect breeding dish, manufactured by SPL in Pocheon, Korea. They were then subjected to a PH_3_ treatment at a concentration of 0.04 mg/L for a duration of 20 h, according to the standards established by the FAO for the PH_3_ resistance test [43]. Due to the knock-down phenomenon, treated insects were examined 72 h after the fumigation was performed. We determined the insecticidal rate by touching each insect’s body with a microscopic needle; if there was no movement observed under a microscope (MDG33, Leica, Wetzlar, Germany), it was determined to be deceased. The trials were conducted three times.

### 2.4. Fumigation Toxicity of Benzoate Compounds against Adults of R. dominica

We performed fumigation treatments with MBe, EB, and VB in 1.5 L glass jars. Different volumes of each tested benzoate (5, 10, 15, and 20 µL) were applied to the inner surface of a piece of parafilm, which was then affixed to the underside of the lid of the jar, creating four tested fumigation concentrations: 5, 10, 15, and 20 µL/1.5 L of air. Plain parafilm without any compound served as the blank control. For both PH_3_-susceptible and -resistant *R. dominica* strains, sets of 20 mixed-sex adults were added to the glass jar. The jars were then tightly sealed to create airtight fumigation chambers. A mortality assessment was conducted following 24 h of exposure. Throughout the exposure time, the glass jars were placed in an incubator under regular controlled conditions (see Section 2.1). Dead insects were those that were on their backs and/or did not move when pressed. The experiment was carried out with five replicates.

### 2.5. Fumigation Toxicity of Benzoate Compounds against Adults of S. oryzae

For *S. oryzae*, we determined the fumigation toxicity of the benzoate compounds based on a procedure described by Yang et al. [44]. In summary, we cut holes in both sides of a 1.5 mL Eppendorf plastic tube for aeration and attached a fine mesh screen to block both holes. Then, 20 mature *S. oryzae* were placed in the tube. Studying the climbing behavior of *S. oryzae*, Plague et al. [45] found that *S. oryzae* adults have a greater propensity than maize weevils to climb upward, so the Eppendorf tubes were used to avoid direct contact between the insects and the treatment chemical. Once the insects were sealed in the tube, it was placed into a 1.5 L glass jar. As described in the previous section, the benzoate treatments were applied to a piece of parafilm to create four fumigation concentrations, 5, 10, 15, and 20 µL/1.5 L air, whereas plain parafilm served as blank controls. The jars were then tightly sealed to be used as fumigation chambers, and mortality was assessed after 24 h of exposure. The experiment was carried out with five replicates. Environmental conditions and mortality assessment procedures were identical to those described in the previous section.

### 2.6. Fumigation Toxicity of Methyl Benzoate against R. dominica and S. oryzae under Feeding Conditions

Based on our findings in the experiments described in Section 3.1 and Section 3.2, we discovered that of the three benzoate compounds, MBe exhibited the greatest fumigation toxicity against all the *R. dominica* and *S. oryzae* strains. So, we conducted fumigant bioassays on PH_3_-susceptible and -resistant *R. dominica* and *S. oryzae* under feeding situations employing varying doses of MBe: 50, 100, 200, and 400 µL of MBe/1.5 L of air. We utilized 50 g of oatmeal or whole rice as food for *R. dominica* and *S. oryzae*, respectively, in 1.5 L glass jars. Twenty mixed-sex adults of *R. dominica* or *S. oryzae* were released into each jar. We then treated filter papers (Whatman No. 1, diameter 10 cm) with the appropriate volume of MBe before placing them in the fumigation chamber. Mortality was assessed after 24 h of exposure for both species, and all treatments were replicated five times.

### 2.7. Statistical Analyses

The adult mortality data of both PH_3_-susceptible and -resistant *R. dominica* and *S. oryzae* were assessed for normality and homogeneity of variance using the Kolmogorov–Smirnov and Levene’s tests, respectively, using the “PROC UNIVARIATE” and “PROC GLM” procedures in SAS 9.4 [46]. Based on the results, we determined that all our data conformed to the normal distribution. We assessed differences between the tested concentrations for each strain of the tested species using one-way ANOVAs followed by Tukey’s post hoc tests (“PROC GLM” in SAS 9.4). However, for each insect species, we used a three-way ANOVA to analyze the interactions between the tested benzoates, concentrations, and strains. For both species, log-probit regression analyses via the “PROC PROBIT” procedure were used to calculate the lethal median concentration (LC_50_) values based on the 24 h mortality data. In addition, significant differences between the strains were determined using 95% confidence intervals (CIs).

## 3. Results

### 3.1. PH_3_ Resistance

We evaluated the PH_3_ resistance rates of PH_3_-susceptible and -resistant strains of two stored-food pest species and found that the PH_3_-susceptible strain of *R. dominica* had a 100% mortality rate, while the PH_3_-resistant strain had a 0% mortality rate. Similarly, the test of the PH_3_-susceptible strain of *S. oryzae* resulted in a death rate of 100%, whereas the PH_3_-resistant strain had a mortality rate of 0%.

### 3.2. Fumigation Toxicity of Benzoate Compounds against PH_3_-Susceptible and -Resistant R. dominica Strains

**Figure 1:**
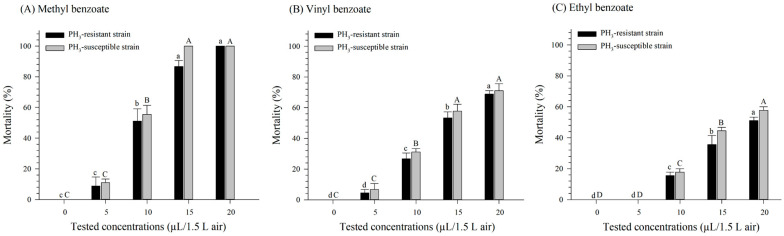
Mortality rates following a 24 h fumigation treatment using methyl benzoate (**A**), vinyl benzoate (**B**), and ethyl benzoate (**C**) against PH_3_-susceptible and PH_3_-resistant strains of *R. dominica*. Values are the means of five replicates. Letters above each bar represent significance groupings for differences between concentrations within each tested strain; means with the same lowercase letter (PH_3_-resistant) or uppercase letter (PH_3_-susceptible) are not significantly different.

Significant differences in mortality were noted between the benzoate compounds and the tested concentrations in both the PH_3_-susceptible and -resistant *R. dominica* strains (*F* = 27.23, *df* = 8, 60, *p* < 0.001) (Figure 1). Against the PH_3_-susceptible *R. dominica* strain, MBe produced the highest mortality (100%) at 15 µL/1.5 L air or higher after 24 h of exposure (Figure 1A), and this rate was significantly higher than those of EB or VB at this concentration. The highest concentration tested, 20 µL/1.5 L air, induced the highest mortality for VB (71.1%) and EB (57.8%) (Figure 1B,C). For PH_3_-resistant *R. dominica*, the highest mortality rates were reached at the highest concentration for all compounds, with significantly higher mortality observed for MBe (100%) than for VB (68.9%) or EB (51.1%) after 24 h of exposure (Figure 1). There were no significant interactions found between the compounds and strains (*F* = 0.11, *df* = 2, 60, *p* = 0.895), concentrations and strains (*F* = 1.65, *df* = 4, 60, *p* = 0.173), or the compounds, concentrations, and strains (*F* = 0.39, *df* = 8, 60, *p* = 0.922) (Figure 1).

The LC_50_ values for MBe, VB, and EB against PH_3_-susceptible adult *R. dominica* were 8.42, 13.72, and 17.03 µL/1.5 L air, respectively, whereas the same values for PH_3_-resistant *R. dominica* were 9.29, 14.45, and 18.97 µL/1.5 L air (Table 1). For both strains, MBe showed more potent fumigation toxicity than VB and EB.

### 3.3. Fumigation Toxicity of Benzoate Compounds against PH_3_-Susceptible and -Resistant S. oryzae Strains

**Figure 2:**
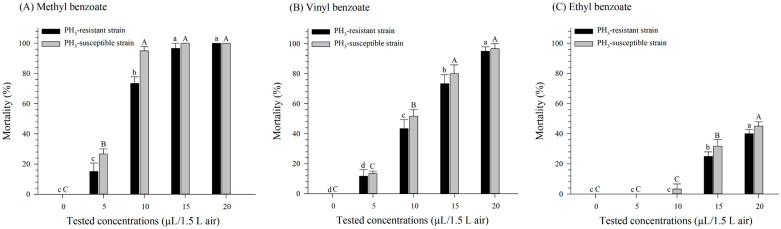
Mortality rates following a 24 h fumigation treatment using methyl benzoate (**A**), vinyl benzoate (**B**), and ethyl benzoate (**C**) against PH_3_-susceptible and PH_3_-resistant strains of *S. oryzae*. Values are the means of five replicates. Letters above each bar represent significance groupings for differences between concentrations within each tested strain; means with the same lowercase letter (PH_3_-resistant) or uppercase letter (PH_3_-susceptible) are not significantly different.

We also observed concentration-dependent mortality against both PH_3_-susceptible and -resistant *S. oryzae*. At the 20 µL/1.5 L air concentration, the mortality rate was at its highest for MBe, VB, and EB in both strains, and a significant interaction between the benzoate compounds and the concentrations was observed (*F* = 65.49, *df* = 8, 60, *p* < 0.001) (Figure 2). Nevertheless, a three-way ANOVA revealed no significant interaction between the strains and the benzoates (*F* = 1.34, *df* = 2, 60, *p* = 0.271) (Figure 2).

The LC_50_ values for MBe, VB, and EB against PH_3_-susceptible *S. oryzae* were 6.04, 9.30, and 20.14 µL/1.5 L air, respectively, whereas the LC_50_ values for PH_3_-resistant *S. oryzae* were 7.60, 10.16, and 21.01 µL/1.5 L air (Table 2). In addition, MBe showed higher fumigation toxicity, and both strains of *S. oryzae* were more susceptible to MBe than *R. dominica*.

### 3.4. Fumigation Toxicity of MBe against PH_3_-Susceptible and -Resistant R. dominica and S. oryzae under Feeding Conditions

**Figure 3:**
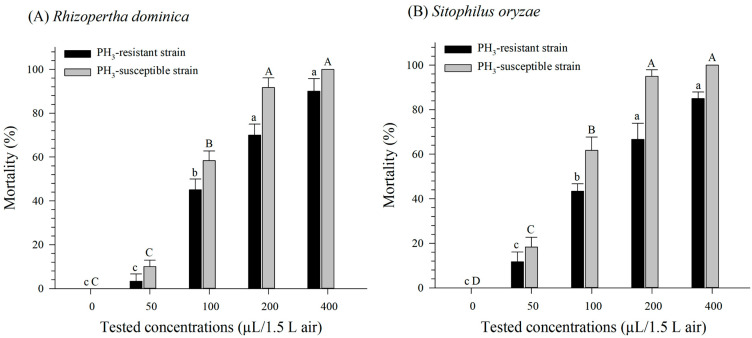
Mortality rates following a 24 h fumigation treatment using methyl benzoate against PH_3_-susceptible and -resistant strains of *R. dominica* (**A**) and *S. oryzae* (**B**) with food available for consumption and protection. Values are the means of five replicates. Letters above each bar represent significance groupings for differences between concentrations within each strain; means with the same lowercase letter (PH_3_-resistant) or uppercase letter (PH_3_-susceptible) are not significantly different.

When a readily available food source was provided, we recorded the highest mortalities at 400 µL/1.5 L air for both strains and species after 24 h of exposure (Figure 3). The highest concentration that was used in the feeding experiment was 20 times higher than that used in the experiments without food. Mortality differed significantly among the tested concentrations for both the PH_3_-susceptible (*F* = 221.97, *df* = 4, 10, *p* < 0.0001) and the PH_3_-resistant strain (*F* = 84.12, *df* = 4, 10, *p* < 0.0001) of *R. dominica* (Figure 3A). While a similar mortality pattern was also observed for *S. oryzae*, no significant differences in mortality between the 200 and 400 µL/1.5 L air concentrations were recorded for either *S. oryzae* strain (Figure 3B), and all the tested concentrations produced significant mortality in both the PH_3_-susceptible (*F* = 157.28, *df* = 4, 10, *p* < 0.0001) and PH_3_-resistant (*F* = 70.11, *df* = 4, 10, *p* < 0.0001) *S. oryzae* strains (Figure 3B).

The LC_50_ value for MBe against PH_3_-susceptible *R. dominica* adults was 93.29 µL/1.5 L air, and for PH_3_-resistant *R. dominica*, it was 134.96 µL/1.5 L air (Table 3). For *S. oryzae*, the LC_50_ values for MBe against PH_3_-susceptible and PH_3_-resistant adults were 83.11 µL/1.5 L air and 134.84 µL/1.5 L air, respectively (Table 3).

## 4. Discussion

This research is the first to assess MBe, VB, and EB as alternative fumigants for PH_3_-resistant populations of the lesser grain borer and rice weevil. Compared to VB and EB, MBe induced high mortality in both strains, both in the absence of food and in the presence of food when applied at high concentrations. Based on our findings with adults, it is possible MBe may be the most toxic gas tested for controlling infestations of these two species. Nevertheless, future work must be carried out on the toxicity of these three compounds against the other life stages of these species, or simply against larger infestations with all life stages present. Overall, *S. oryzae* exhibited a higher susceptibility to MBe than *R. dominica* for both susceptible and resistant strains, with and without a food source. However, LC_50_ values for phosphine-susceptible strains were lower than those for phosphine-resistant strains in both species. This phenomenon may arise when greater amounts of hydrocarbons in the cuticle of resistant insect strains play an important role in inhibiting fumigant penetration into the insect bodies [47]. This reduces fumigant toxicity in general since no pesticide can act until it reaches its target in the insect’s body [47,48].

In a previous study, Morrison et al. [36] demonstrated the fumigation toxicity of MBe against a susceptible strain of *R. dominica* with and without food. The findings indicated that the highest concentration of MBe (30.88 mg/L) had significant effects on the survival of *R. dominica* adults during a 24 h exposure period. In our study, we discovered similar patterns of toxicity in adults with or without food. The direct fumigation effects of MBe on PH_3_-susceptible and -resistant strains of *R. dominica* and *S. oryzae* were significantly reduced when even very small amounts of food were present. Overall, the presence of food increased the LC_50_ values of MBe by 11–14-fold for both strains of *R. dominica*, whereas for *S. oryzae*, the LC_50_ values of MBe were 13–17-fold higher when food was present. Like other aerosolized liquids and modified atmospheres used to control insects in stored products, e.g., ozone [49], MBe could not penetrate the commodity very effectively. Additionally, the presence of food may assist individuals in detoxifying MBe through metabolic and genomic pathways that have not yet been determined. Previous research has frequently documented the rapid recovery of stored product insects following exposure to insecticides when food was available (for example, as described in Arthur [50]). The effectiveness of many methods for managing stored-item pests, such as chemical control strategies like fumigants, aerosols, and grain protectants, is greatly impacted by the presence of food [36].

In resistance-control programs at food facilities, many alternative fumigants have recently been used. According to Opit et al. [51], sulfuryl fluoride has shown high levels of fumigation toxicity against all life stages of PH_3_-resistant *R. dominica* and *Tribolium castaneum* (Herbst). Furthermore, it has the potential to be used for the control of PH_3_ resistance. In another study, ethyl formate at 70 and 90 mg/L exhibited high fumigation toxicity (100% mortality) against the eggs, early larvae, and adults of a PH_3_-resistant strain of *S. oryzae* [42]. In addition, Sakka et al. [52] reported complete parental mortality of *S. oryzae* at low oxygen (1%) levels. However, all these alternative fumigants have shown fumigation toxicity only in the absence of food, while in our results, MBe showed strong fumigation toxicity both in the absence of food and when food was available. 

The effectiveness of MBe against insects that are protected within products may be improved in the future via the development of new methods. To illustrate this point, Fulcher et al. [53] suggested that a thermal fogger might be used to improve the dispersive capability of MBe fumigation during a specific period. Additionally, aerosols are sometimes utilized in postharvest commodities [54,55]. Aerosolizers are responsible for the release of liquid pesticides into a food facility in the form of extremely tiny droplets, in the order of several microns. While aerosols and fumigants are generally considered separate methods, some chemicals may possess characteristics that make them suitable for both categories. For example, when MBe is in liquid form, it demonstrates direct contact and ingestion toxicity against a variety of insect pests [26,27,56,57]. Furthermore, it has fumigant characteristics that have been evaluated in a variety of studies [28,30,36,37]. It is possible that aerosolizing MBe would increase its penetration throughout the space of a food facility, enabling the compound to concurrently perform the functions of a fumigant and a direct contact insecticide (aerosol) for those individuals who are on the surface of a commodity.

Methyl benzoate has the capacity to affect the neurological systems of animals [58,59]. Mostafiz et al. [60] discovered that a low concentration (LC30 = 0.22%) of MBe significantly reduced (by 65%) the activity of acetylcholinesterase (AChE) in *Aphis gossypii* Glover (Hemiptera: Aphididae). Also, recent studies revealed that MBe, when used as a fumigant against adult *Acanthoscelides obtectus* Say (Coleoptera: Chrysomelidae) and *Musca domestica* Linnaeus (Diptera: Muscidae) larvae, significantly reduced AChE activity [61,62]. The main route of a fumigant’s entrance into the insect body is through the respiratory system [63]. For example, volatile substances interfere with breathing and inhibit metabolic activity in insects, resulting in physiological and biochemical changes that induce hypoxia and insect mortality [61]. 

The development of alternative fumigants for insects that are found in food facilities involves concerns aside from effectiveness. Specifically, food facility managers need a chemical that is economically viable and does not leave any unpleasant smells on stored items, and they must be open to adopting this new solution. The present study did not specifically investigate the economic aspects of MBe usage or the sensory characteristics of the final goods made from MBe-treated commodities, and it is recommended that future research explore these aspects, possibly providing more evidence to support the use of this molecule. Moreover, the development of combination fumigation techniques that include PH_3_ and MBe may be helpful in addressing PH_3_ resistance in insects.

## 5. Conclusions

There has been a significant increase in the number of insect populations that are resistant to PH_3_ all over the world. Additionally, PH_3_ continues to play an important role in the global postharvest supply chain. As a result, stakeholders are likely to be willing to use an alternative fumigant if it is effective, not prohibitively expensive, and does not impinge on the quality of the commodity. We tested the effectiveness of MBe against both PH_3_-susceptible and -resistant populations of *R. dominica* and *S. oryzae*, and the results demonstrate that it has the potential to be an effective alternative fumigant against both populations. Verifying the validity of the other two requirements of an alternative fumigant will be crucial in the future, particularly if MBe has the potential to be a specialized product for controlling *R. dominica* and *S. oryzae*. Nevertheless, more investigation is necessary to evaluate and advance the natural evaporation of MBe in large-scale experiments, enhance control effectiveness, and provide guidelines for treatments using MBe on a commercial scale.

## Figures and Tables

**Table 1:** Median lethal concentration (LC_50_) values for three benzoate compounds against PH_3_-susceptible and -resistant *R. dominica* strains after a 24 h fumigation treatment.

Species	Benzoate Compounds	Strains ^†^	LC_50_ (µL/1.5 L Air)	95% CI (Lower–Upper)	Slope (±SEM)	*X*^2^ (*df*)
*Rhyzopertha dominica*	Methyl benzoate	PH_3_-S	8.42	-	6.52 (1.52)	16.68 (2)
		PH_3_-R	9.29	(6.33–12.11)	5.67 (0.77)	5.82 (2)
	Vinyl benzoate	PH_3_-S	13.72	(12.60–15.03)	3.73 (0.38)	0.44 (2)
		PH_3_-R	14.45	(13.17–16.06)	3.41 (0.37)	0.52 (2)
	Ethyl benzoate	PH_3_-S	17.03	(15.69–18.84)	4.01 (0.51)	2.65 (2)
		PH_3_-R	18.97	(17.23–21.67)	4.01 (0.52)	1.85 (2)

^†^ PH_3_-S = susceptible strain; PH_3_-R = resistant strain; CI = confidence interval; *df* = degrees of freedom.

**Table 2:** Median lethal concentration (LC_50_) values for three benzoate compounds against PH_3_-susceptible and -resistant *S. oryzae* strains after a 24 h fumigation treatment.

Species	Benzoate Compounds	Strains ^†^	LC_50_ (µL/1.5 L Air)	95% CI (Lower–Upper)	Slope (±SEM)	*X*^2^ (*df*)
*Sitophilus oryzae*	Methyl benzoate	PH_3_-S	6.04	(5.64–6.46)	7.65 (0.78)	0.15 (2)
		PH_3_-R	7.60	(7.04–8.15)	6.00 (0.49)	1.26 (2)
	Vinyl benzoate	PH_3_-S	9.30	(8.55–10.04)	4.52 (0.37)	3.36 (2)
		PH_3_-R	10.16	(6.62–13.88)	4.39 (0.63)	5.64 (2)
	Ethyl benzoate	PH_3_-S	20.14	(18.53–22.77)	5.30 (0.76)	4.09 (2)
		PH_3_-R	21.01	-	6.29 (1.75)	6.51 (2)

^†^ PH_3_-S = susceptible strain; PH_3_-R = resistant strain; CI = confidence interval; *df* = degrees of freedom.

**Table 3:** Median lethal concentration (LC_50_) values for methyl benzoate against PH_3_-susceptible and -resistant strains of *R. dominica* and *S. oryzae* after a 24 h fumigation treatment in which food was available for consumption and protection.

Tested Compound	Species	Strains ^†^	LC_50_ (µL/1.5 L Air)	95% CI (Lower–Upper)	Slope (±SEM)	*X*^2^ (*df*)
Methyl benzoate	*Rhyzopertha dominica*	PH_3_-S	93.29	(85.16–101.91)	4.52 (0.39)	1.08 (2)
		PH_3_-R	134.96	(47.98–319.46)	3.09 (0.58)	10.19 (2)
	*Sitophilus oryzae*	PH_3_-S	83.11	(75.37–91.14)	4.25 (0.38)	0.38 (2)
		PH_3_-R	134.84	(117.66–154.09)	2.38 (0.22)	2.64 (2)

^†^ PH_3_-S = susceptible strain; PH_3_-R = resistant strain; CI = confidence interval; *df* = degrees of freedom.

## Data Availability

No supplementary data are available; all data are contained in the manuscript.
